# Research collaboration in Tehran University of Medical Sciences: two decades after integration

**DOI:** 10.1186/1478-4505-7-8

**Published:** 2009-04-22

**Authors:** Reza Majdzadeh, Saharnaz Nedjat, Jaleh Gholami, Sima Nedjat, Katayoun Maleki, Mostafa Qorbani, Mostafa Shokoohi, Mahnaz Ashoorkhani

**Affiliations:** 1 School of Public Health, Centre for Academic and Health Policy (CAHP), TUMS-KTE Study Group, Tehran University of Medical Sciences, Tehran, Iran; 2 Centre for Academic and Health Policy (CAHP), TUMS-KTE Study Group, Tehran University of Medical Sciences, Tehran, Iran; 3 School of Medicine, Golestan University of Medical Sciences, Golestan, Iran; 4 Physiology Research Center, Kerman University of Medical Sciences, Kerman, Iran

## Abstract

**Background:**

In 1985 medical schools were integrated into the Ministry of Health, and the Ministry of Health and Medical Education was created in Iran. Under this infrastructure education, research and service provision are unified, and it is expected that collaboration between researchers and decision makers become easier in such an integrated context.

The question here is how the researchers behavior in the biggest medical university of the country towards collaboration is, i.e. how much do decision makers participate in different stages of research? Which factors affect it?

**Methodology:**

The samples under study were all Tehran University of Medical Sciences (TUMS) completed research projects that had gotten grants in 2004 and were over by the time this study was done. Two questionnaires were designed for this study: i) the research checklist which was filled for 301 projects, ii) the researcher's questionnaire, which was sent to principle investigators, 208 of which were collected. Multiple linear regression analysis was used for evaluating the potential factors affecting individuals 'collaboration score'.

**Results:**

Only 2.2 percent of TUMS' projects initiated in 2004 have had collaboration as a joint PI or co-investigator from non-academic organizations. The principle investigators mean collaboration score was 2.09, where 6 was the total score. So the collaboration score obtained was 35%. The 'type of research' had significant association with the collaboration score which is shown in the linear regression; collaboration was seen more in clinical (*p *= 0.007) and health system researches (*p *= 0.001) as compared to basic research.

**Conclusion:**

The present study shows that not many individuals collaborated as co-investigators from outside the university. This finding shows that research policy makers need to introduce interventions in this field. And assessment of barriers to collaboration and its facilitating factors should be considered in order to make it actually happen.

## Background

"Cooperation between researchers and decision makers throughout the research process i.e. choosing the research topic up to its implementation, and not just securing grants is considered as collaboration in research". Collaboration in research is one of the ways which strengthens the possibility of utilizing research findings [1-3]. One of the solutions put forth in confronting the barriers to knowledge translation in policy making is strengthening ties between researchers and policy makers for maintaining long-term connections and commitment [4]. This connection is recognized as a predicting indicator for the increased uptake of research results [5].

The advantages of decision makers' collaboration at different levels of 'research conduction', 'researcher' and 'decision maker' have also been studied in the literature. The following points were noted: research results are made useful, access to data sources are facilitated, decision makers can be involved in the research process, expert opinions within the decision makers organization become accessible, the researchers become familiar with the decision makers environment, researchers' satisfaction is guaranteed in securing grants not validated in the academic environment, decision makers become more familiar with their own activities, decision makers' research skills increase, decision makers become aware of other ongoing research activities and become familiar with researchers' perspectives [6]. For these reasons many studies have been conducted to examine collaboration among users and its extent at different levels of research [6] and/or its effect on knowledge transfer [7,8].

In a systematic review conducted on decision makers perceptions on their use of evidence three factors were said to have a positive impact on it: maintaining personal contact, timeliness and the mode of presentation of materials. The barriers were noted to be: un-timeliness, irrelevant research topics, and lack of trust and power in securing budget [9]. Structures that can strengthen interactions between researchers and decision makers can reduce barriers and increase the benefits of collaboration in research [10].

The main feature of Iran's health system is the integration of medical universities into Ministry of Health in 1985 which led to the formation of 'Ministry of Health and Medical Education' (MOHME). Under this infrastructure, health education and research are the responsibility of the same ministry that provides services. Such a structure can create close ties between academics and decision makers. Are there any traces of collaboration in research between academics and decision-makers twenty years after integration? How much do researchers and decision makers collaborate in different stages of research? And which factors affect it?

In the past few years scientific publications in medicine has considerably increased in Iran and in the early 90s its scientific production has had the greatest growth in the Middle East region [11]. Also, the numbers of articles that have been published in ISI journals has doubled from 1997 to 2001 [12]. Tehran University of Medical Sciences (TUMS) holds 12% of the country's medical academic members. In its assessment in 2003 up to its last assessment in 2007 MOHME declared that TUMS had gained the first rank in research among medical universities in the country [13].

Therefore, in this context, three objectives were in mind: What is the extent of collaboration? How much collaboration takes place at different stages of research? And which academic factors affect it? The study will help health research policy makers realize how much collaboration exists in the integrated context.

## Methodology

### Population under study

The population under study was 'completed research projects' granted in 2004 and over by 2006. Two sources of data have been identified from this population:

#### a) Proposals and final reports

The number of research projects that possessed the inclusion criteria of this study was 315, 301 of which were available and whose data collection forms were filled (95.5% of all the projects).

#### b) Principle investigators (PIs)

The researcher's questionnaire was sent to the projects' PIs and eventually 208 questionnaires were collected. Of the remaining, 32 individuals were inaccessible and 75 did not respond in spite of being mailed thrice (response rate was 66%).

The research proposals of PIs who had not responded were compared with those who had responded to assess selection bias. This was done by reviewing the 'problem statement ' of the research proposals. We observed that 24% of the individuals who hadn't responded to the questionnaire had mentioned choosing their topics on the basis of needs assessment. The difference between these two was statistically insignificant (*p *= 0.17).

### Data collection tools

They consisted of two sections: a) Data collection form b) Researcher's questionnaire. (See additional files 1 and 2 respectively).

a) The data collection form was filled using available documents (proposals and final reports). These resources were used to extract the particulars of project PIs and co-investigators including their specialty and working location, the type of research, amount of funds, and funders.

b) Researcher's questionnaire (which was a self-administered one) was sent to the PI thrice at the most and at one month intervals. After literature review the content validity of the questionnaire was approved and reviewed by a panel of experts. Thereafter the pre-test was done for evaluating the feasibility; face validity and reliability, i.e. a pilot was done of 10 projects' data collection forms by studying 10 projects and creating necessary changes. Also, 20 researchers filled the questionnaire twice at two week intervals to assess the repeatability and internal consistency of the questions. The intra-class correlation which was considered as the repeatability indicator was 0.69 to 0.72 in the domains under study. The internal consistency (Cronbach's alpha) of these domains was 0.63 to 0.76.

The questionnaire included the following domains: the time participants allocated to research activities, the reason of choosing the research topic and collaboration of the end-users of research at different stages of the project. These stages included all levels from choosing the research topic to dissemination of research results. Thereafter, a single score was given to each of these activities. Their total was then considered as their collaboration score and also as a dependent variable for studying the effect of other variables (range: 0–6).

### Data analysis

Description of the population under study, their collaboration and the relationship between independent variables with the collaboration score were studied through statistical tests including frequency, chi-square, analysis of variance and multiple linear regression analysis. SPSS 11.5 software was used. The 'collaboration score' is the sum of collaboration items from 'choosing a research topic' to 'dissemination of the results to research users'. The dependent variable in the regression analysis was the collaboration score. The independent variables were: researcher's sex, academic rank, professional record, tenure status, having executive responsibility, type of research, method of choosing the research topic and percent of total time allocated to research.

### Ethical considerations

This study was approved by the ethics review board as part of TUMS research projects reviewing process.

## Results

### Population under study

#### Proposals and final reports

The review of 301 research proposals shows that the total budget of the projects under study was a little less than 1'290'000 US $, whereas in only 20 projects (6.6%) part of the cost had been provided by organizations outside the university. The total budget gained for these projects from outside the university was a little over 86'000 US $, approximately 6.7% of the total budget spent on the projects.

#### Principle investigators

Two hundred and eight PIs participated, 130 of which were male (62.5%). The minimum age was 25, the maximum 72, and the mean age was 45.6 years (SD = 9.4). Regarding their professional status 15% of individuals were non-academic members, 7% were instructors; 33, 26 and 19 percent were assistant professors, associate professors and professors respectively. Among these, 181 individuals (87%) worked full-time and 10 (4.8%) worked part-time. Their professional record (number of years working as a professional) ranged from 1 to 43 years and the mean number of working years in the university was 14.3 (SD = 8.5). Aside of education and research 123 individuals had executive responsibilities such as management of a hospital, school, department or ward, research deputy of the school and/or research center etc. Seventy two individuals (34.6%) were involved in education and/or research alone.

The researchers were divided into three groups according to the projects under study into basic sciences (n = 46), clinical (n = 101) and health system researchers (n = 61).

According to the PIs claims, comparison of time allocated to research in these three groups shows that the mean time allocated to research in basic science researches was 41% (sd = 22%), and a significant difference (*p *< 0.001) existed between the two groups of clinical and health system researches (HSR) respectively: 27% (sd = 16%) and 30% (sd = 19%). Researchers were asked about their 'method of choosing the research topic'. Thirty one participants (14.9%) stated their personal interest or repeating others researches to be the sole reason. This percentage was 23.9 in the basic sciences, 7.9 in the clinical and 19.7 in the health system researchers.

Table 1 shows the response given by researchers to the question of 'reason of choosing the research topic'. The most frequent response given by basic science researchers (47.8%) was that 'this project was part of a series of projects in response to a specific question'. In clinical science researchers it was in response to needs felt in clinical decision making (69.3%), but in health system researchers the most frequent response was personal interest (55.7%). It is necessary to consider that researchers could choose more than one option at the same time in this question. HSRs had been conducted on public executive organizations' demand more often than the other researches and this difference was significant (*p *= 0.001). The important point is that few researches have been conducted in response to private sectors request (such as pharmaceutical and medical equipment companies).

**Table 1:** TUMS researchers' 'reasons of choosing the research topic' during 2004, based on the type of research

	**Basic**		**Clinical**		**Health**		
		
**Reason of choosing the research topic**	**Number** n = 46	**Percent** 22.1	**Number** n = 101	**Percent** 48.6	**Number** n = 61	**Percent** 29.3	**P-value**
I was personally interested in this topic	16	34.8	50	49.5	34	55.7	0.09

Reviewing other researches tempted me to take on this subject and repeat the research project	11	23.9	34	33.7	15	24.6	0.31

I carried out this research in response to questions brought about in other research projects	15	32.6	41	40.6	11	18.0	0.01

This project is part of a series of research projects that have been carried out to answer a specific question	22	47.8	40	39.6	25	41.0	0.64

This project is needed by one of the public executive organizations and has been carried out upon their demand	1	2.2	6	5.9	21	34.4	0.001

This project is needed by non-governmental organizations or centers (like pharmaceutical and medical instrument companies) and has been carried out upon their demand	1	2.2	3	3.0	1	1.6	0.86

I chose this title through inspecting the needs of managers and policy-makers	1	2.2	10	9.9	21	34.4	0.001

I chose this title through inspecting the needs of practitioners in decision-making	13	28.3	70	69.3	6	9.8	0.001

### Collaboration in research

Table 2 shows the result of the question on 'research users' collaboration: 'choosing the research topic' converted to the dichotomous variable in which 'doing research with collaboration' was considered one level (last four rows), and the first four rows -which were considered as choosing the research topic without collaboration. Among the different stages of research, collaboration was seen most in choosing the research topic. Collaboration in choosing the research topic was 30.4%, 74.3% and 60.7% in basic science, clinical and HSRs respectively. Fifty one researchers stated the users had not collaborated in any kind of activity (24.5% of 208 cases).

**Table 2:** Collaboration with research users at various stages of research, according to the type of research

	**Basic (n = 46)**		**Clinical (n = 101)**		**Health (n = 61)**	
	**Number**	**Percent**	**Number**	**Percent**	**Number**	**Percent**

Choosing the research topic	14	30.4	75	74.3	37	60.7

Design of the objective and methodology of the project	11	23.9	45	44.6	36	59.0

Execution of the project	8	17.4	26	25.7	30	49.2

Analysis and interpretation of research results	4	8.7	27	26.7	16	26.2

Preparation of reports	7	15.2	24	23.8	11	18.0

Dissemination of the research results	7	15.2	23	22.8	18	29.5

No collaboration	20	43.5	21	20.8	10	16.4

Number of collaborations: Mean*(sd)	1.19 (1.33)		2.25 (1.53)		2.47 (1.60)	

* Based on the analysis of variance done the mean number of cases of collaboration in basic sciences had significant differences with clinical and HSR groups with a *P *< 0.001 in both cases. This is such that the *P *of the two groups (clinical and HSR) is almost 0.38.

For obtaining the overall result of collaboration each case of collaboration was given one score. The minimum score was zero and the maximum was 6. The mean 'collaboration score' was 2.09 (sd = 1.58) i.e. 35% of the sum score. The mean 'collaboration score' in HSR (2.47) was higher than the others. Analysis of variance also showed that the difference between these three groups was significant (*p *= 0.001). Two by two comparisons showed that the mean 'collaboration score' in basic sciences was significantly lower than the other two groups.

### Factors related to collaboration in research

The 'collaboration score' was considered as a dependent variable of linear regression. All other variables that have been introduced so far were considered as independent variables. The result of the regression analysis is illustrated in table 3. On the basis of comparison with 'basic science researchers, 'the variables that have shown to have a significant relationship include 'clinical researchers' and 'health system researchers'. That is, as compared to basic science researchers, clinical researchers raised the sum score by 0.92 (*p *= 0.007), and health system researchers raised the sum score by 1.17 (*p *= 0.001).

**Table 3:** The result of influential factors on number of collaborations based on regression analysis with the score of collaboration as a dependent variable

	**Regression coefficient**	**Standard deviation**	**P-value**
Sex (male/female)	-0.11	0.25	0.66

Professor (in comparison to an assistant professor)	0.00	0.36	0.99

Associate professor (in comparison to an assistant professor)	0.66	0.30	0.32

Instructor (in comparison to an assistant professor)	0.20	0.48	0.68

Non-academic member (in comparison to an assistant professor)	0.45	0.49	0.36

Tenure status (full time/half time)	-0.60	0.62	0.34

Professional record (years)	-0.03	0.02	0.09

Executive responsibility (has/hasn't)	-0.23	0.25	0.37

Time allocated to research (percent of total time)	0.00	0.01	0.64

Clinical researches (in comparison to basic science researches)	0.92	0.34	0.007

Health researches (in comparison to basic science researches)	1.17	0.36	0.001

Constant coefficient	2.44	0.95	0.01

## Discussion

The main findings of this study are summarized as the following three points: a) Only 28 and 5 projects (13.5% and 2.4% respectively) were done on the demand of public and private organizations respectively (table 1). b) 2.2% of the university's projects have been done in collaboration with other organizations, c) Only 6.6% of the university's projects had been funded by organizations from outside the university (including public and private).

While interpreting these findings it must be kept in mind that there is a possibility of information bias in study subjects where the researcher's questionnaire was the data collection tool. Even though in the beginning of this study the questionnaire went through the reliability test and was recognized as reasonably reliable, we believe that there may be some information bias because of some questions whose answers may be considered as undesirable by the researchers. In case of data collection tools which are based on project proposals and reports the possibility of this error is less.

Regarding collaboration itself the following must be taken into consideration: In a study Ross et al examined some important factors such as 'levels of decision makers' collaboration in the research topic'. Based on this study the levels of collaboration are divided into three forms: *A) Formal supporter*- in this condition the decision makers are not actively involved in the research process. They are unaware of the ongoing efforts being made, but support the research objectives, legalize the implementation of research and facilitate access to resources. *B) Responsive audience*- the decision maker is active to the extent that he responds to the researcher's ideas, provides the necessary information and gives consultations. And finally *C) Integral partner*- where the decision maker is completely involved in the research process [6]. Therefore even though names of individuals have been mentioned in research proposals or in final reports we cannot be sure of their extent of collaboration. Is this collaboration merely limited to having his/her name mentioned in the list, or has he/she really collaborated?

In recent years efforts have been made to connect academics to decision makers in Iran. Three types of efforts may have affected the findings of this study:

i. Integration of medical universities into Ministry of Health,

ii. Encouraging collaborative research through joint financial support,

iii. Allocation of financial resources to applied research.

i. Even though initially the aim of integration was to increase the capacity of medical students' admission and to strengthen community-oriented medical education, nonetheless the unified management of universities and health executive organizations could have brought researchers and decision makers closer to each other.

ii. On the basis of legislations in the country's five-year *'Economic, Social and Cultural Development Plan'*, if there is an executive organization, public or private, who'd be willing to secure 40% of the financial resources of a research project for a research institute (and/or university) the remaining funds will be secured by the government [14].

The current study in Iran shows that, practically, this sector does not contribute to a considerable portion of the research funds allocated. Considering the fact that the studies of this research were defined in 2004 which was the last year of the *'Third Development Program'*, it is evident that a fair share of these resources has not been allocated to TUMS.

iii. In 2003 the board of trustees in TUMS agreed to allocate 2% of the financial resources to applied research (these were part of HSR projects that were aimed at solving the university's problems). This share increased to 5% of funds in 2006. Perhaps because this policy exists in the university's budget there is a significant difference (*p *= 0.001) between health system researchers' reason of choosing the research topic and the other researchers. They had the highest percentage (34.4%) of 'choosing their research topic' on executive organizations' demand which is shown in table 1. As illustrated in Table 2, the type of research, including clinical and HSRs, show a significant relation with the collaboration score as compared to basic science researches.

In spite of these interventions, existing data at national level show that only 3–6% of research resources are secured by the private sector (including private companies, scientific associations and non-governmental organizations) [12].

If we look at collaboration generally at all levels of research, Table 2 shows that in one fourth of all cases there was no collaboration between researchers and research users at different stages of design, implementation, data analysis, production and dissemination of research results. The most common collaborative effort was 'choosing the research topic', which is consistent with earlier studies [15].

Therefore merely introducing interventions at the structural level of decision making organizations will not guarantee collaboration between researchers and decision makers. No doubt, collaboration in research is not a simple task. Basically the working atmosphere of researchers and decision makers is not the same. It is believed that rarely high quality evidence-based processes exist that have been presented to decision makers in a short period of time [16].

Some of the barriers were known to be: not prioritizing research according to policy makers' needs, inadequate time, and lack of trust in research results [17]. In another qualitative study conducted in TUMS, the factors affecting knowledge translation (and indeed collaboration) in the same integrated context have been divided into individual (researcher and decision maker) level and the context of exchange [18]. When publishing articles is given more importance in academic members' promotion criteria than solving decision makers' problems, it is no surprise that collaboration is not practiced by researchers [19]. The prerequisite for collaboration is having a common vision on both sides. As a result executive organizations will utilize the research results more and will try to contact researchers themselves [10]. It has been proven how much Health Technology Assessment agencies' vision of collaboration affects their knowledge translation behaviors [8]. That's why creating networks between decision makers and their collaboration in research can have obvious effects on research based decision making [20].

In fact one of the factors that can make collaboration happen is building trust between researchers and decision makers. However it must be noted that it takes time to build this trust and it won't happen overnight. It will happen with continuous group work [21]. MacLeod tells about her experiences in the article and believes collaboration between researchers and decision makers to be a result of six rules: *"engaging the right decision-makers, determine what's in it for you and for them, develop a sustained relationship, live in their world once in a while, think of doing research differently and build integrative research infrastructures" *[22].

So in order to create change and have collaboration take place the prerequisites must be met. The current study shows even in an integrated context the level of collaboration may not be enough. In addition to the structure, other factors should be taken into account and conditions should be made favorable for promoting collaboration in Iran.

## Conclusion

The present study shows that the projects of TUMS don't have much collaboration with organizations outside the university. Even though the current structure in Iran (i.e. MOHME's stewardship over medical universities) creates the opportunity for researchers and decision makers to collaborate but barriers and facilitators should be considered in order to make it actually happen.

Interventions such as 'creating knowledge networks and valuing exchange efforts' may be helpful in strengthening the context of collaboration.

## Abbreviations

HSR: Health system research; MOHME: Ministry of Health and Medical Education; PI: Principle Investigator; TUMS: Tehran university of Medical Sciences.

## Competing interests

The authors declare that they have no competing interests.

## Authors' contributions

All authors contributed equally to this manuscript.

## Supplementary Material

Additional File 1 **Research checklist**. The checklist provided is the data collection form which was filled using proposals and final reports. Click here for file

Additional File 2 **Researcher's questionnaire**. The researcher's questionnaire was filled by the principle investigator of the research. Click here for file
