# Supplementary material for: Research collaboration in Tehran University of Medical Sciences: two decades after integration
Source: Health Res Policy Syst. 2009 Apr 22;7:8. doi: 10.1186/1478-4505-7-8 (PMC2679006; doi:10.1186/1478-4505-7-8)
Supplement: Additional File 1 — Research checklist. The checklist provided is the data collection form which was filled using proposals and final reports. [file 1478-4505-7-8-S1.doc]

**Research Checklist**

Please use only the material documented in the proposals and final reports for completing the form, and refrain from writing your personal deductions.

1. Project code:
2. Project title:
3. Principle Investigators’ information:

|  | A | B | C | D |
| --- | --- | --- | --- | --- |
| Row | Name and Surname | Last degree | Field of study | Working location |
| 1 |  |  |  |  |
| 2 |  |  |  |  |
| 3 |  |  |  |  |
| 4 |  |  |  |  |

1. Co-investigators’ information:

|  | A | B | C | D |
| --- | --- | --- | --- | --- |
| Row | Name and Surname | Scientific degree | Field of study | Type of co-operation |
| 1 |  |  |  |  |
| 2 |  |  |  |  |
| 3 |  |  |  |  |
| 4 |  |  |  |  |
| 5 |  |  |  |  |
| 6 |  |  |  |  |
| 7 |  |  |  |  |
| 8 |  |  |  |  |
| 9 |  |  |  |  |
| 10 |  |  |  |  |
| 11 |  |  |  |  |

1. Please specify the type of research:
2. Clinical ❑ ii. Basic ❑ iii. Health System Research ❑

**Introduction and the relevant part of the Proposal**

1. Has the project been conducted in collaboration with a specific center or organization?
2. Yes ❑ ii. No ❑
   1. If yes, which organization? ………..

**Introduction**

1. Has the project been conducted on a specific organization’s demand?
2. Yes ❑ ii. No ❑
   1. If yes, which organization? ………..
3. Is it related to other projects? (Is it part of a series of related projects?)
4. Yes ❑ ii. No ❑
5. What was the reason (mentioned in the introduction) the researcher choose this title for the project?
6. It was a review of others’ research and repetition of their projects. ❑
7. It was in response to questions brought up in other research projects. ❑
8. It was part of a series of research projects that have been carried out in response to a specific question. ❑
9. One of the executive organizations needed and demanded it. ❑

9.iv.a) The organization’s name………..

1. This project was needed by non-governmental organizations and/or centers (like pharmaceutical and medical equipment companies) and has been carried out upon their request. ❑
2. Through inspecting managers and policy-makers’ needs. ❑
3. Through inspecting clinicians’ needs in decision-making. ❑
4. Others ❑ 9.viii.a. Please specify ………..………..
5. Unclear ❑
6. Has the introduction mentioned which groups can benefit from the results of this study?

You may choose more than one option.

1-People **❑ 2-** Special groups of the society **❑ s** 3-Patients **❑**

4-Health managers and policy makers **❑**

5-Managers and policy makers of other organizations **❑** 6-Other researchers **❑**

7-Healthcare providers (clinical, laboratory, health etc) **❑**

8-Others ❑ 10.8.a. Please list the names ……….. 9-Unclear **❑**

**Type of study**

1. Please specify the type of study.

1- Case series **❑** 2- Cross sectional **❑**  3- Case control **❑** 4- Cohort **❑**

5- Interventional and/or Clinical trial **❑** 6- Experimental **❑**

7- Studies for manufacturing drugs or medical equipment **❑**

8- Inaugurating a scientific implementing system **❑** 9- Test examinations **❑**

10- Method examinations **❑** 11- Qualitative studies **❑**

12- Health system managerial studies **❑** 13- Software design **❑**

**Objectives**

1. Have the target audiences of the project been addressed in the practical objectives?
2. Yes ❑ ii. No ❑

**Methodology and Table of Co-investigators**

1. Has the target audiences collaboration been mentioned in the design, methodology or data analysis?
2. Yes ❑ ii. No ❑ iii. The executives and co-investigators are considered as target audiences of the project ❑

**Funds**

1. Have funds for research-based knowledge transfer activities been considered in the budget section?
2. Yes ❑ ii. No ❑
3. How much? ………..
4. How much is the project’s total budget? ………..
5. Has part of the budget been supplied by another organization or center?
6. Yes ❑ ii. No ❑

If yes: 17.1. Which organization? ……….. 17.2. How much? ………..

1. Was the project an academic thesis? i. Yes ❑ ii. No ❑

**Final report**

**Discussion and Conclusion**

1. Have the target audiences been clearly addressed in the project report or its executive summary? i. Yes ❑ ii. No ❑
2. Has a clear practical suggestion been made to the target audience in the project report?
3. Yes ❑ ii. No ❑

If yes, does this suggestion state by whom, how, in which field, and what measure should be taken?

20.1. By whom? i. Yes ❑ ii. No ❑

20.2. How? i. Yes ❑ ii. No ❑

20.3. In what field? i. Yes ❑ ii. No ❑

20.4. What measure? i. Yes ❑ ii. No ❑

20.5. Please explain ………..

1. Has a clear practical suggestion been made to the target audience in the executive summary?
2. Yes ❑ ii. No ❑

If yes, does this suggestion state by whom, how, in which field, and what measure should be taken?

21.1. By whom? i. Yes ❑ ii. No ❑

21.2. How? i. Yes ❑ ii. No ❑

21.3. In what field? i. Yes ❑ ii. No ❑

21.4. What measure? i. Yes ❑ ii. No ❑

21.5. Please explain ………..

Questionnaire completed by: ………..………..

Date of completion: ………..………..
