# Supplementary material for: Research collaboration in Tehran University of Medical Sciences: two decades after integration
Source: Health Res Policy Syst. 2009 Apr 22;7:8. doi: 10.1186/1478-4505-7-8 (PMC2679006; doi:10.1186/1478-4505-7-8)
Supplement: Additional File 2 — Researcher's questionnaire. The researcher's questionnaire was filled by the principle investigator of the research. [file 1478-4505-7-8-S2.doc]

**Researcher's Questionnaire**

Personal Information:

1. Sex: 1) male ❑ 2) female ❑
2. Year of birth.…
3. Professional status:

i) Non-academic board member ❑ ii) Instructor ❑

iii) Assistant Professor ❑ iv) Associate Professor ❑

v) Professor ❑ vi) Research expert ❑

vii) Educational expert ❑ viii) Duty General Physician ❑

ix) Medical Resident ❑ x) PhD Student ❑

xi) Master's Degree Student ❑ xii) Other ❑ (please mention details…)

1. How many years of service have you had with your most recent degree? .............
2. Tenure status: 1) Full time ❑ 2) Part time ❑
3. Please define the approximate percentage of your time that you've spent on each of the responsibilities mentioned below in the past two years (education and research mean teaching and investigating respectively. Please leave other executive responsibilities for education and research for the part on executive activities.)

|  | **Activity** | **percentage**  Please consider that the sum of percentages equals 100 |
| --- | --- | --- |
| 1 | Education (could be associated with provision of clinical services) |  |
| 2 | Research |  |
| 3 | Executive responsibilities |  |
| 4 | Clinical or laboratory services only |  |
| 5 | Other (please name) |  |
|  | **Sum** | 100 |

1. Which of the following factors led to the choice of the title for this research project? (You can choose more than one option)
2. I was personally interested in this title. ❑
3. Reviewing other researches tempted me to take on this subject and repeat the research project. ❑
4. I carried out this research in response to questions brought about in other research projects. ❑
5. This project is part of a series of research projects that have been carried out to answer a specific question. ❑
6. This project is needed by other organizations (other than ours) and/or non-governmental centers (like pharmaceutical and medical equipment companies) and has been carried out upon their demand. ❑
7. I chose this title through inspecting the needs of managers and policy-makers. ❑
8. I chose this title through inspecting the needs of practitioners in decision-making. ❑
9. Other ❑ Please specify………………………………………………..
10. Which of the following activities have you carried out for interacting and collaborating with research users in this project? (In case you choose an option, shortly explain the manner of your interaction)
11. Interaction with research users in the design of the objective and the methodology of the project ❑

In which way…………………………………..……………………..

1. Interaction with research users in the implementation of the project ❑

In which way…………………………………………………………

1. Interaction with research users in the analysis and interpretation of research results

In which way…………………………………………………………

1. Interaction with research users in the production of research results (such as reports, articles, etc) ❑

In which way……………………………………………………………

1. Interaction with research users in the dissemination of the research results (for publication purposes in journals and/or presentation in mass media and/or conferences) ❑

In which way……………………………………………………………

1. Neither ❑
2. To which target groups have you attempted to transmit the results of this research? (you can choose more than one option)
3. People or Patients ❑
4. Health managers and policy makers ❑
5. Healthcare providers (clinical, laboratory, health, etc) ❑
6. There is no need to transmit the results of this research ❑
7. Other ❑ (please name)……………….
8. Neither ❑
9. Please explain the activities that you have carried out in this regard.
10. To which target groups have you 'not' attempted to transmit the results of this research? (in spite the fact that they were targets of the project)
11. People or Patients ❑
12. Health managers and policy makers ❑
13. Healthcare providers (clinical, laboratory, health, etc) ❑
14. There is no need to transmit the results of this research ❑
15. Other ❑ (please name)……………….
16. Neither ❑

1. Which of the following activities have you done in regard to your research project (by research users we mean all target audiences who can benefit from the research results).

| Row | Activity | I haven’t done it because | | |  | | |
| --- | --- | --- | --- | --- | --- | --- | --- |
|  |  | 1. I didn’t think it was necessary | 2. The means were not available | 3. It wasn’t my duty | 4. I tried to do this but was successful | 5. I’ve done it once | 6. I’ve done it more than once |
| 1 | Sending the complete report of the research project to users | 1 | 2 | 3 | 4 | 5 | 6 |
| 2 | Sending a summary report of the project to users | 1 | 2 | 3 | 4 | 5 | 6 |
| 3 | Writing an article of the research project and its publication in domestic journals | 1 | 2 | 3 | 4 | 5 | 6 |
| 4 | Writing an article of the research project and its publication in international journals | 1 | 2 | 3 | 4 | 5 | 6 |
| 5 | Provision and sending texts compatible with users' language (such as simple texts for patients or special texts for managers or practical reports for clinical or lab colleagues, special reports for industrial fellows or academicians) | 1 | 2 | 3 | 4 | 5 | 6 |
| 6 | Displaying the results on the web site to allow stakeholders access | 1 | 2 | 3 | 4 | 5 | 6 |
| 7 | Mailing or emailing articles, reports, or summaries for stakeholders without their request | 1 | 2 | 3 | 4 | 5 | 6 |
| 8 | Mailing or emailing articles, reports, or summaries for users upon their request | 1 | 2 | 3 | 4 | 5 | 6 |
| 9 | Presenting research results in conferences, seminars, and domestic meetings | 1 | 2 | 3 | 4 | 5 | 6 |
| 10 | Presenting research results in conferences, seminars, and international meetings | 1 | 2 | 3 | 4 | 5 | 6 |
| 11 | Presenting results to reporters, radio and TV for dissemination in the media and participation in interviews | 1 | 2 | 3 | 4 | 5 | 6 |
| 12 | Printing research results in non-scientific publications (such as journals or newspapers in which, the general public is interested) | 1 | 2 | 3 | 4 | 5 | 6 |
| 13 | Other cases (please explain)……. |  |  |  | 4 | 5 | 6 |

In case "zero" demonstrates inactivity and 10 is the sign of great activity in transfer of knowledge produced through research, which number will you choose to score your activities in knowledge transfer in this specific research project?

(zero to ten) ………………………………………………….
